# Synergistic enhancing-memory effect of donepezil and S 47445, an AMPA positive allosteric modulator, in middle-aged and aged mice

**DOI:** 10.1007/s00213-017-4792-5

**Published:** 2017-11-22

**Authors:** S. Bretin, A. Krazem, N. Henkous, C. Froger-Colleaux, E. Mocaer, C. Louis, N. Perdaems, A. Marighetto, D. Beracochea

**Affiliations:** 1 Institut de Recherches Internationales Servier, Pôle d’Innovation Thérapeutique Neuropsychiatrie, Suresnes, France; 2 Institut de Neurosciences Cognitives et Intégratives d’Aquitaine (INCIA), Université de Bordeaux, UMR CNRS 5287, Allée Geoffroy Saint-Hilaire, Bat B2, 33613 Pessac, France; 3 Froger-Colleaux C, Porsolt Research Laboratory, Z.A de Glatiné, 53940 Le Genest-Saint-Isle, France; 4 Institut de Recherches Servier, Pôle d’Innovation Thérapeutique Neuropsychiatrie, Croissy-Sur-Seine, France; 5 Pôle Expertise en Pharmacocinétique, Orléans, France; 6 INSERM, Neurocentre Magendie, Physiopathologie de la plasticité neuronale, U1215, 33077 Bordeaux, France

**Keywords:** Aging, Amnesia, Acetylcholine, Glutamate, AMPA receptors, Alzheimer disease

## Abstract

Positive allosteric modulators of AMPA receptors (AMPA-PAMs) are described to facilitate cognitive processes in different memory-based models. Among them, S 47445 is a novel potent and selective AMPA-PAM. In order to assess its efficacy after repeated administration, S 47445 effect was evaluated in two aging-induced memory dysfunction tasks in old mice, one short-term working memory model evaluated in a radial maze task and one assessing contextual memory performance. S 47445 was shown to improve cognition in both models sensitive to aging. In fact, administration of S 47445 at 0.3 mg/kg (s.c.) reversed the age-induced deficits of the working memory model whatever the retention interval. Moreover, in the contextual task, S 47445 also reversed the age-induced deficit at all tested doses (from 0.03 to 0.3 mg/kg, p.o.). Since donepezil, an acetylcholinesterase inhibitor, induces only moderate symptomatic effects on memory in Alzheimer’s disease patients, an alternative strategy for treatment of cognitive symptoms could be to act simultaneously on both glutamatergic AMPA receptors and cholinergic pathways by combining pharmacological treatments. The present study further examined such effects by assessing combinations of S 47445 and donepezil given orally during 9 days in aged C57/Bl6J mice using contextual memory task (CSD) and the working memory model of serial alternation task (AT). Interestingly, a significant synergistic memory-enhancing effect was observed with the combination of donepezil at 0.1 mg/kg with S 47445 at 0.1 mg/kg p.o. in the CSD or with S 47445 at 0.1 and 0.3 mg/kg in AT in comparison to compounds given alone and without any pharmacokinetic interaction.

**Electronic supplementary material:**

The online version of this article (10.1007/s00213-017-4792-5) contains supplementary material, which is available to authorized users.

## Introduction

The role of acetylcholine is now well established in cognition processes (for review, see Hasselmo 2006) since the blockade of muscarinic cholinergic receptors by scopolamine impairs the encoding of new memories (Atri et al. 2004) whereas the activation of nicotinic cholinergic receptors by cholinergic agonists enhances the encoding of new information (Levin et al. 2006).

Since neurodegenerative processes impacting cholinergic pathways were reported in the brains of Alzheimer’s disease (AD) (Bartus et al. 1982; Davies and Maloney 1976), acetylcholinesterase inhibitors (as donepezil) which inhibit the breakdown of acetylcholine were initially developed and remain the standard treatment of cognitive impairment in AD. Nowadays, targeting other pharmacological pathways becomes nevertheless essential to develop more effective drugs for AD (Hampel et al. 2011; Herrmann et al. 2011).

The ionotropic glutamate AMPA (α-amino-3-hydroxy-5-methyl-4-isoxazole propionic acid) receptors are highly expressed in the mammalian central nervous system. These receptors mediate fast excitatory synaptic transmission and facilitate expression and maintenance of long-term potentiation (LTP), a form of synaptic plasticity that is believed to underlie memory formation (Palmer et al. 2005; Whitlock et al. 2006; Wollmuth and Sobolevsky 2004). The involvement of AMPA receptors in different aspects of memory was therefore widely discussed (Robbins and Murphy 2006; Lynch and Gall 2006). Further, positive allosteric modulators of AMPA receptors (AMPA-PAMs) have been developed more particularly in the last 10 years (Morrow et al. 2006; Ward et al., 2010; Pirotte et al., 2013). They were shown to slow desensitization of AMPA receptors and enhance synaptic excitatory currents, thereby promoting synaptic transmission and plasticity (Black 2005; Morrow et al. 2006; Lynch and Gall 2006; O’Neill and Dix 2007; Partin 2015). They were reported to facilitate episodic and spatial working memories in a number of behavioral studies in rodents, monkeys, and humans, both memories known to be early impaired in AD patients (Bernard et al. 2010; Black 2005; Morrow et al. 2006; Lynch and Gall 2006; O’Neill and Dix 2007; Partin 2015).

S 47445 is a potent and selective AMPA-PAM (Bretin et al. 2017; Giralt et al. 2017). Studies have shown that S 47445 rescues in vivo CA3-CA1 long-term potentiation and structural synaptic changes in middle-aged mice and displayed neurotrophic effects in dorsal hippocampus and prefrontal cortex of aged rats (Calabrese et al. 2017). It also presented procognitive effects as S 47445 displayed dose-related improvement in both episodic-like memory as observed in the object recognition in rats and in working-like memory in the spontaneous alternation task in normal adult mice after an acute administration (Bretin et al. 2017).

Moreover, acting on either glutamate or acetylcholine seems to be valuable strategies to enhance cognitive functions and to create a synergistic effect. Indeed, combined treatments can be more effective than compounds alone and can allow using lower doses of each compound, i.e., minimizing the potential negative side effects.

Many rodent behavioral tasks have been designed to potentially reproduce different aspects of the disrupted cognition in AD in particular the episodic and working memories. By using contextual and temporal cues, contextual serial discrimination (CSD) allows to study simultaneously contextual declarative (episodic-like) memory versus spatial semantic (reference) memory in middle-aged mice (Béracochéa et al. 2007, 2s008; Célérier et al. 2004). Further, middle-aged mice in this task display episodic-like memory deficits but not spatial memory deficits, a selectivity that may represent an early form of age-related deficit in AD. The most widely used paradigms for working memory are maze type tasks (Webster et al. 2014). Marighetto et al. (2008a, Marighetto et al., 2008b) developed a model of aging-related decline in short-term/working memory in a radial maze. This model allows studying two main components of working memory, i.e., the short-term maintenance (retention) and the organization of information both deteriorated in aged mice (Mingaud et al. 2008; Tronche et al., 2010). Such deficits are also observed in aged animals in a sequential alternation task requiring to alternate successive spatial choices over a series of trials (Vandesquille et al. 2013).

Therefore, the aim of the present study was to investigate (i) the procognitive impact of S 47445 chronically administered alone either orally or subcutaneously in a CSD task or in a radial arm maze working memory task and (ii), according to the results drawn in experiment 1, to determine the potential synergistic procognitive effects of the combinations of the S 47445 with the acetycholinesterease inhibitor, donepezil using the CSD and serial alternation task (SA).

## Material and methods

### Animals

Male mice of the C57Bl/6J inbred strain obtained from Charles River (France) have been used for the CSD and SA tasks. Upon arrival, animals remained in collective cages before experimentation began. They were housed individually at least 10 days before behavioral testing. Mice were 14–15 months old (“middle-aged”) for testing in the CSD task or 18–19 months old (“aged”) for testing in the sequential alternation task in experiment 2. All animals had continuous access to food and water and were placed on a 12-h light-dark cycle (8:00 a.m.–8:00 p.m.) in a temperature-controlled room (22 ± 1 °C). All test procedures were conducted during the light phase of the cycle.

For the radial arm maze task, male C57Bl/6 mice (Janvier labs, Le Genest Saint Isle, France) aged either 3–5 months (young mice) or 22–24 months (aged mice) at the beginning of the experiments were used. Mice were individually housed 2 weeks before testing. Food deprivation was introduced progressively to maintain each animal around 90% of its ad libitum weight along testing. All test procedures were conducted during the light phase of the cycle.

Ethical statement: “Principles of laboratory animal care” were followed and experiments have been performed in accordance with the European Communities Council Directive 2010/63/UE and the local ethical committee for animal experimentation. Animals were sacrificed by cervical elongation immediately after either behavioral testing or plasma sampling.

### Drugs

S 47445 micronized form was synthetized by Servier, Suresnes, France. Donepezil, polysorbate 80 (Tween80®), and hydroxyethyl cellulose were obtained from Sigma-Aldrich, Lyon, France. S 47445 was suspended in 1% (*w*/*v*) hydroxyethyl cellulose and 1% (*v*/*v*) polysorbate 80 in distilled water for oral administration (p.o.) or in 1% (*w*/*v*) hydroxyethyl cellulose and 1% (*v*/*v*) polysorbate 80 in saline (NaCl 0.9%) for subcutaneous (s.c.) administration.

For subcutaneous administration, donepezil was diluted in saline (0.9% of NaCl). The doses of S 47445 and donepezil are expressed as free base. For Irwin test, donepezil (Sequoia Research Products Ltd) was dispersed in 1% (*w*/*v*) hydroxyethylcellulose and 1% (*v*/*v*) polysorbate 80 in distilled water.

Treatments were administered per os daily for nine consecutive days for studies involving the CSD and SA tasks. The 8th and the 9th oral administrations were given 60 min before acquisition and test phases in the CSD task, or before the training and the test sessions in the SA task.

In the radial arm maze task, treatments were administered subcutaneously (s.c.) once a day 60 min prior to testing from the 2 days of habituation to the 12 days of memory testing.

### Behavioral tasks

#### Contextual serial discrimination task in 14–15-month-old mice (experiments 1 and 2)

**Fig. 1:**
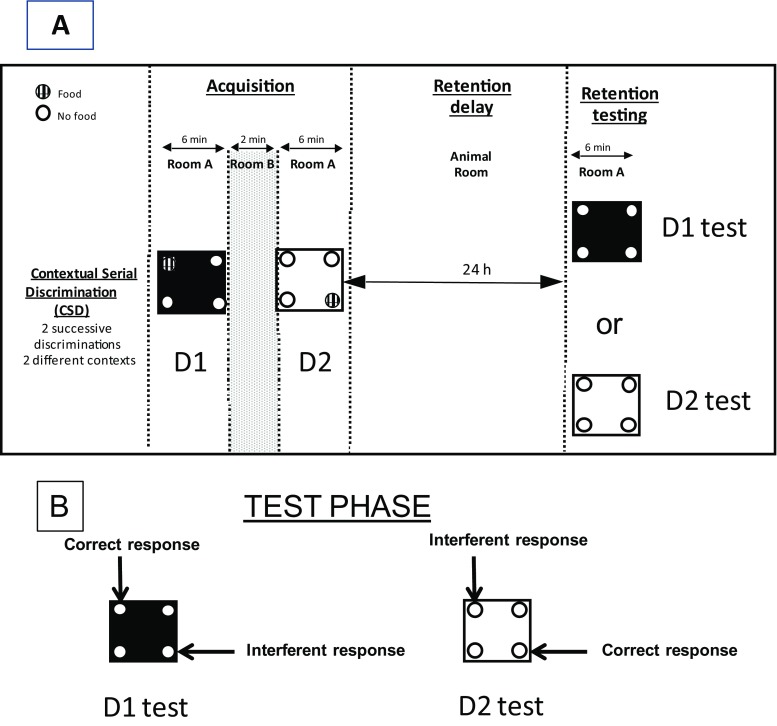
**a** Contextual serial discrimination: at the acquisition phase, mice performed two consecutive spatial discrimination sessions varying by the color and texture of the floor, i.e., D1, discrimination 1, and D2, discrimination 2. For each discrimination session, only one out of the four holes of the apparatus was baited (hashed circles). The two discrimination sessions were separated by a 2-min delay interval during which animals were placed in room B. A 24-h delay was interpolated between the acquisition and the test phases, during which mice were returned in the colony room. One hour prior to acquisition and test phases, mice received a per os administration of the compounds or vehicle solution in a chamber placed in a room (room C) different from the one in which the behavioral experiments were conducted (room A). Subsequently, mice were submitted to the test phase in which they were repositioned on the floor of the first discrimination without any food pellet in the apparatus. **b** Two types of responses were calculated: (i) correct responses corresponding to head-dips into the hole baited at the acquisition of the first discrimination (D1), on the same floor context; (ii) interference responses corresponding to head-dips into the hole baited at the other (second) discrimination (D2). Both parameters allow calculation of the SCM score

The studies were carried out on mice according to the procedure described in full by Celerier et al. (2004) and Tronche et al. (2010) (Fig.1). Briefly, the mouse learns two consecutive spatial discriminations (D1 and D2) in a four hole-board apparatus (45 × 45 × 30 cm) enclosed with gray Plexiglas walls. The floor of the hole-board was interchangeable (white and rough versus black and smooth). On the floor, four holes opening on a food cup (3-cm diameter × 2.5-cm depth) were located 6 cm away from the sidewalls. Photocells located into the holes allowed an automatic recording of the number of head-dips in each hole.

Mice were partially food-deprived for 1 week prior to experimentation and were maintained on a 12 ± 2% loss of their body weight throughout behavioral testing to increase motivation for food reward. During the food deprivation phase, mice were handled daily to familiarize them with the experimenter.

During the acquisition session, mice were first placed at the center of the board in a plastic tube for 15 s and then learned two successive spatial discriminations (D1 and D2) for 6 min each and were each separated by a 2-min interval. Between two sessions, each mouse was placed in its home cage in an animal housing room. For D1, ten 20-mg pellets were available only in one of the four holes in the board. For D2, ten 20-mg pellets were located in the opposite symmetrical hole. The environmental spatial cues remained at the same place for D1 and D2. Within each group, the floors used for the first and second discrimination (D1 and D2) were counterbalanced at the acquisition phase from one mouse to the other. A minimal amount of eight pellets should be eaten per mouse at either D1 or D2 during the acquisition phase.

The retention test was carried out 24 h after the acquisition phase. During memory testing, D1 and D2 discriminations were performed in independent groups of mice, being replaced either on the floor of D1 or of D2. The food-deprived mice were replaced either on the floor of D1 or on the floor of D2. In each case, mice were allowed to explore the apparatus freely and performance was assessed by measuring the exploration for each hole for 6 min without any pellets in the apparatus. During the procedure, photocells allowed measurement of the number of head dips in each hole. Different parameters were further calculated: % of correct responses (head-dips into the hole previously baited on the same floor context), % of interfering responses (head-dips into the hole previously baited on the other floor context), and % of errors (head-dips into the two holes never previously baited). These parameters allowed calculus of the “strength” of contextual memory” score (SCM) (% correct responses − % interference responses). Since correct responses are based on the use of the internal context (color of the floor) and interfering responses are based on the use of spatial allocentric cues previously associated with discrimination on the other floor, thus the trend of SCM score toward a positive difference represents the gain of internal contextual memory, at the expense of allocentric spatial one. SCM scores were already used to assess the effects of age and pharmacological treatments on memory processes (Sors et al. 2017).

The effects of donepezil (0.03, 0.1, and 0.3 mg/kg) and S 47445 (0.03, 0.1, 0.3, and 1.0 mg/kg) on memory in the CSD task were determined in independent groups of 12 mice each.

Fig. 1 Protocol of the contextual serial discrimination

#### Radial maze working memory task in aged mice (22–24 months old) (experiment 1)

This task consists in “continuous concurrent serial alternation” challenging short-term memory of successive arm visits of mice in a radial maze and models everyday-like memory (Al Abed et al. 2016). The short-term retention of variant information and the organization of information in short-term memory to reduce interference can be evaluated (Mingaud et al. 2008).

**Fig 2:**
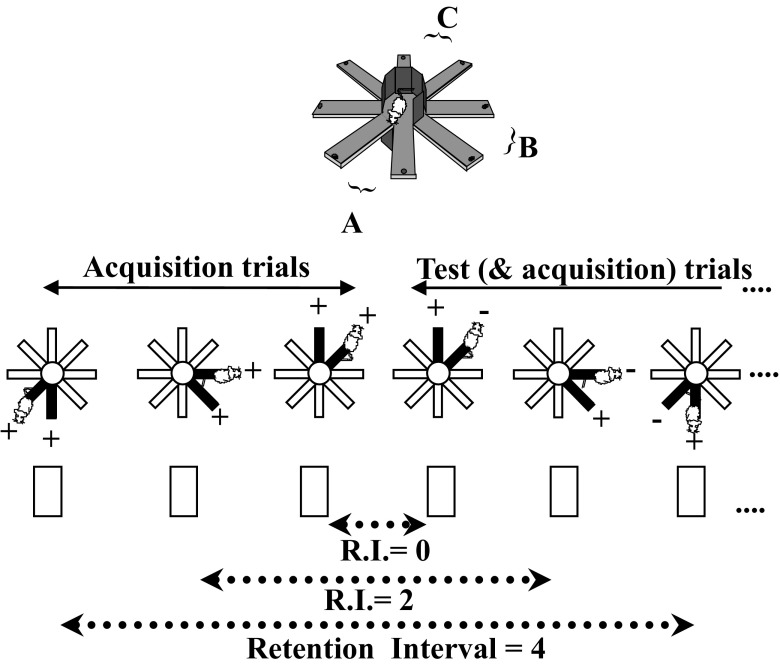
The radial maze working memory task. Each session consisted of alternate presentations of pairs A, B, and C. The first three trials with each pair were acquisition trials (both arms rewarded). Each of the following trials was at the same time an acquisition trial (for the following trial with the same pair) and a test trial (for the arm visit made during the preceding trial with the same pair); the rewarded arm was the one that has not been visited on the preceding trial with the same pair. Memory demand varied across successive trials according to the retention interval RI (i.e., the number of intervening trials with other pairs between two successive presentations of the same pair). The retention interval for one specific arm visit (e.g., within pair A) depends on the number of concurring trials (i.e., with pairs B and C) occurring till next trial with the same pair (A). Indeed, such a variable amount of interposed trials proportionally increases the delay between acquisition and test and may also generate retroactive interference. There are five levels of retention interval: from 0, corresponding to “no interposed pair,” to 4, corresponding to a sequence of four interposed trials between n-1 and n trials.

The procedure is described in Figure 2.. The radial maze (Imetronic system, Pessac, France) was fully automated and enabled by video-tracking automatic testing and recording. Briefly, each mouse was separately assigned to six adjacent arms which were grouped into three pairs (A, B, C). These three pairs were presented repeatedly among the successive trials of each testing session. The location of the food reward varied in each pair according to an alternation rule among successive trials. Namely, among the two arms opened in a specific trial, the food was inserted in the arm which was not visited in the previous trial within the same pair. The mouse must therefore remember which of the two arms was visited in a specific trial until next presentation of the same pair, and so on in order to alternate its choice among successive presentations of any pair. At the beginning of each daily session, the mouse was placed in the central platform of the radial maze with all arms closed. One pair of arms only was opened at the beginning of each trial, and the mouse was required to visit one arm. The door of the non-chosen arm was closed as soon as the mouse reached the end of the chosen arm. The mouse was allowed to run back to the central platform of his own accord, and then, the door of the chosen arm was closed. The mouse remained in the central platform until the following trial (intertrial interval 10 s). Since three pairs were concurrently used, three specific arm visits must be kept in memory by the mouse until their utilization by using short-term working memory. After 2 days of free exploration in the maze (habituation), mice were submitted to 12 consecutive testing sessions: one session of 23 trials (3 “acquisition-only trials,” one by pair, and 20 “acquisition and test trials”) per day, i.e., a total of 276 trials (240 test trials).

Memory performance was investigated in six parallel groups of mice as follows: (1) young vehicle-treated), (2) aged vehicle-treated, (3) S 47445 at 0.1 mg/kg, (4) S 47445 at 0.3 mg/kg, (5) S 47445 at 1 mg/kg, and (6) donepezil at 0.3 mg/kg.

During habituation, the time needed for visiting all six arms per session day was measured. During memory testing, memory performance was evaluated according to the percentage of correct alternation responses between successive trials with the same pair in three different manners. Firstly, overall performance was calculated as a mean percentage over the 12 sessions of training or 12 days (of 20 test trials each). Secondly, performance as a function of retention interval (RI) was also calculated: for one specific arm visit (e.g., within pair A), RI depends on the number of concurring trials (i.e., with pairs B and C) occurring till next trial with the same pair (A). There were five levels of retention interval: from 0, corresponding to “no interposed pair,” to 4, corresponding to a sequence of four intervening trials between n-1 and n trials. Performance was expected to decrease as retention interval increases. Thirdly, performance is expected to be modulated by proactive interference due to irrelevant memories of preceding trials. The assessment of proactive interference was conducted across trials involving the same arm pairs. In particular, the level of proactive interference was dependent of the number of intervening trials between the to-be remembered trial, n-1 and the interfering trial (n-2): the smaller the number of trials between n-1 and n-2, the higher is the proactive interference. The mean percentage of correct choices over the 12 sessions of training was calculated for trials with low proactive interference (low PI) and high proactive interference (high PI), respectively.

#### Sequential alternation working memory in 17–18-month-old mice (experiment 2)

The spontaneous alternation (SA) task was performed in independent groups of mice as regards to the CSD and radial maze tasks. The SA task allows investigation of aged-induced working memory impairments (Vandesquille et al. 2011). In this task, mice are submitted to a series of successive choices in a T-maze. To alternate from trial to trial, animals have to remember at each choice (except the first one) only the previous trial and to reset the interfering information from trial to trial over a series. This resetting mechanism is a main aspect of working memory (Aggleton et al. 1986; Béracochéa and Jaffard, 1987; Kesner 2005).

Behavioral tests were conducted in a gray Plexiglas T-maze. Stem and arms are 35 cm long, 15 cm wide, and 10 cm high. The starting box (10 cm × 12 cm) and each goal arm are separated from the central alley by a vertical sliding door, with opening and closing monitored by a computer. Photoelectric cells allow recording of both the choice of the goal arm (left or right) and the latency (in seconds) that elapsed between the opening of the starting box and the closing of the goal box. The T-maze is located at the center of a room with various allocentric cues (white or black or striped card boards) located on the wall 1 m above the apparatus.

Animals were not food deprived, and no food reinforcement was used. Animals were first submitted to a habituation phase consisting of two free exploration sessions of 10 min each, over two consecutive days (one session per day) in the apparatus with all doors opened. At the end of the habituation phase, all animals were submitted to a training phase, involving a series of seven successive trials separated by a 30-s intertrial interval (ITI). The training phase was aimed at familiarizing the animals with the opening and closing of the doors.

For the training session, animals were submitted to a sequence of seven successive identical trials. At the beginning of a trial, mice were placed in the starting box and after a confinement period (30 s, predefined ITI), the door to the stem was opened. When the mouse entered one of the goal arms, the door was closed and the chosen arm was recorded. After a 30-s confinement period in the chosen arm, the mouse was gently returned to the starting box for a second trial, identical to the first one. To avoid olfactory cues in the apparatus, visible traces of urine were removed and the general procedure was implemented as in the training phase, except that the ITI was extended to 90 s and animals were submitted to a series of eight successive trials. This ITI was chosen according to a previous study showing that, over such a delay, aged mice of 17–18 months were dramatically impaired compared with young adult mice (4–5 months old), whereas no difference was observed at shorter delays (i.e., 5 and 30 s) (Vandesquille et al. 2011, 2013). Moreover, an 8th trial was added to the series which was separated from the 7th one by a 5-s ITI. Indeed, any improvement of SA rates at this shorter delay would indicate that aging, treatments, and the repetition of trials within a series did not modified the motivation to alternate and, therefore, that any SA impairments on the first trials of the series rely on a memory loss and not on a motivational impairment. The alternation score (in percentage) was used as an index of memory performance. An alternation is defined as a visit in a given arm followed by a visit into the other arm. The successive sequence of visits for the seven first trials determines the level of alternation. A percentage of alternation was calculated: number of alternations performed/number maximal of alternation. This percentage is calculated for each mouse.

#### Irwin study in mouse

The effect of S 47445 after high doses administration was assessed on general behavior in mice using the primary observation (Irwin) test (Irwin 1968). S 47445 and donepezil were first evaluated alone at 3, 10, 30, and 100 mg/kg p.o. and 0.3, 1, 3, and 10 mg/kg p.o., respectively (four mice/group). S 47445 and donepezil were then evaluated in combination at 3, 10, 30, and 100 mg/kg p.o. and 0.3, 1, 3, and 10 mg/kg p.o., respectively (four mice/group). Combination experiments were performed in four separate tests corresponding to each of the four doses of donepezil, combined with vehicle or with one of the four doses of S 47445. Male Rj: C57BL/6J mice (Elevage Janvier, 53940 Le Genest-Saint-Isle, France), 23–27 g body weight range, were stabilized for at least 5 days after delivery in macrolon cages in groups of 10 (housing period) (25 × 19 × 13 cm) and then of 4 (starting the day before the test and during the test) in macrolon cages (25 × 19 × 13 or 32 × 13 × 14 cm) on wood litter with free access to food and water. This study was performed at Porsolt Research Laboratory under Good Laboratory Practice.

Mice were administered the test substance and were observed in simultaneous comparison with a control group given vehicle (non-blind conditions). Between one and three treated groups were compared with the same control at any one time. All animals within a treatment group were observed simultaneously. For assessment of the combination, mice received S 47445 doses 30 min before administration of donepezil. In order to keep the same conditions, all the mice receiving a single treatment also received another administration with vehicle. The observations was done at the following time points: T0 (S 47445 or vehicle administration); T0 + 15 min, T0 + 30 min, T0 + 45 min, T0 + 60 min, T0 + 90 min, T0 + 120 min, T0 + 150 min, T0 + 180 min, T0 + 210 min, T0 + 24 h.

Behavioral modifications, physiological and neurotoxicity symptoms, and rectal temperature were recorded according to a standardized observation grid derived from that of Irwin. The grid contains the following items: death*, convulsions*, tremor*, Straub tail*, altered activity, excitation, jumping*, abnormal gait* (rolling, tip-toe), motor incoordination*, altered muscle tone, loss of grasping, akinesia, catalepsy, loss of traction, writhing*, piloerection*, stereotypies* (sniffing, chewing, head movements), head-twitches*, scratching*, altered respiration*, aggression*, altered fear, altered reactivity to touch, ptosis, exophthalmia, loss of righting reflex, loss of corneal reflex, analgesia, defecation/diarrhea, salivation, lacrimation, and rectal temperature (hypothermia/hyperthermia).

The symptoms marked (*) were both observed continuously for 15 min after S 47445 administration and/or after donepezil administration and at the respective time points indicated above.

### Pharmacokinetics study

As the two compounds were administered simultaneously in experiment 2, it was important to assess a potential pharmacokinetic interaction which could have faked a synergistic effect by increasing the blood exposure**.** For the pharmacokinetics study, an additional day of treatment (day 10) was done after the CSD test session for blood sampling to measure concentrations of S 47445 in blood and donepezil in plasma, on the combinations groups where either the most important synergistic effect was observed, or containing the highest doses of S 47445 and donepezil. However, as samples of the active doses used in the pharmacological experiments were below the limit of quantification for S 47445 and for donepezil, it was decided to assess interaction using a higher dose of donepezil (1.0 mg/kg) in combination with S 47445 at 0.3 mg/kg. Accordingly, blood sampling (250 μL/sample) was performed on three groups: S 47445 (0.3 mg/kg) and donepezil (1.0 mg/kg); combination of donepezil (1 mg/kg) and S 47445 (0.3 mg/kg) with at least *n* = 4 per sampling time.

The sampling times were 30 min, 1, 2, 4, and 6 h.

Sampling for determination of S 47445 blood concentration was done by taking 10 μL of blood from lateral caudal vein of mice into sodium heparinized capillary tubes, and blood was spotted onto DMPK-A cards (Whatmann,GE Healthcare, France). Then, the cards were dried at room temperature for at least 2 h and S 47445 analysis was determined using ultra-high-performance liquid chromatography with tandem mass spectrometric detection assay (LC/MS-MS) (Covance Laboratories Ltd., England). For donepezil, two time points of blood sampling were performed in each mouse. Plasma was extracted from the blood sample by centrifugation (+ 4 °C, 1500*g*, 10 min) and stored at − 80 °C. Then, plasma samples were sent in dry ice to Celerion (Switzerland) for the pharmacokinetic analysis. The concentrations of donepezil were determined in plasma using liquid chromatography and mass spectrometry detection. The limit of quantification was 1 ng/ml for S 47445 and 0.1 ng/ml for donepezil.

### Statistical analyses

For the CSD task, behavioral data were analyzed by one-way or two-way factorial analyses of variance, followed when adequate, by post hoc (Dunnett’s test) comparisons using the least significant difference test, with a *p* < 0.05 statistical threshold using SAS v9.2 software (SAS Institute, Cary, NC). The young vehicle group was used to assess the effects of aging on SCM score but was excluded from analyses of the drug effects on memory in middle-aged animals.

For the radial maze working memory task, behavioral data (percentage of correct responses) were analyzed using an ANOVA followed by Fisher PLSD’s post hoc tests. Independent ANOVAs were performed to analyze the effect of age (young versus aged mice vehicle-treated) and to analyze the effect of either treatment in aged mice (S 47445 or donepezil versus vehicle). These ANOVAs were also used for analyzing retention interval (5 levels, 0–4) and proactive interference (2 levels: low, high). Analyses were performed using SAS v9.2 software (SAS Institute, Cary, NC).

For the sequential alternation task, mean and standard errors of the mean (SEM) were calculated for each group of treatment. In addition to % alternation drawn over the seven trials of the series, behavioral performances were also expressed in percentage of alternation drawn from blocks of two consecutive trials (block 1: trials 2 + 3; block 2: trials 4 + 5; block 3: trials 6 + 7). The analyses per trial blocks allowed evaluation of proactive interference over the series (Vandesquille et al. 2011, 2013). Statistical analyses were performed using the Statview 5.0 software. Data were analyzed by one-way or two-way factorial analyses of variance, followed, whenever adequate, by post hoc comparisons using the least significant difference test, with a *p* < 0.05 statistical threshold. A one-sample *t* test was used for comparison with chance level (i.e., 50%) and a Student’s *t* test for between two group comparisons.

## Results

### Experiment 1: determination of active and subactive doses of donepezil and S 47445 administered alone on age-induced memory decline mice models

#### Contextual serial discrimination in middle-aged mice (14–15 months old)

**Fig. 3:**
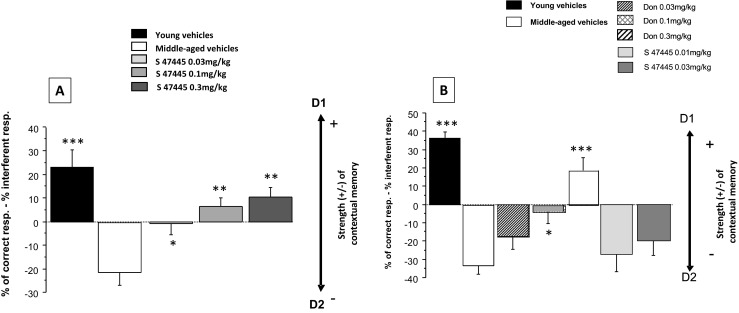
Effects of S 47445 and donepezil administered alone on SCM scores in middle-aged mice. Part A: SCM scores are expressed as means ± SEM. SCM scores are obtained through calculation (% of correct responses − % of interfering responses). Middle-aged vehicle-treated mice exhibit a significant decrease of SCM scores as compared to young vehicle-treated mice (*p* < 0.001); the three S 47445 doses significantly improved performance as compared to middle-aged vehicle-treated mice, the greater memory-enhancing effect being observed with the higher S 47445 dose (0.3 mg/kg). Part B: as can be seen, middle-aged vehicle-treated mice exhibit a significant decrease of SCM scores as compared to young vehicle-treated mice, thus confirming results of part A. The intermediate (0.1 mg/kg) and the highest donepezil doses (0.3 mg/kg), but not the lower one (0.03 mg/kg) significantly increased SCM scores as compared to middle-aged vehicle-treated mice; in contrast, the two doses of S 47445 (0.01 and 0.03 mg/kg) did not significantly improved performance (NS in all comparisons with vehicles). *p < 0.05; **p < 0.01; ***p < 0.001 as compared to middle-aged vehicles

The first experiment was divided in two studies, A and B. In study A (Fig. 3a), the effects of S 47445 (0.03, 0.1, and 0.3 mg/kg p.o.) were studied on memory performance (SCM scores) as compared to vehicle (*N* = 12 mice in each group). The study B was aimed at determining the effects of a lower range of doses of S 47445 (0.01 and 0.03 mg/kg p.o.) and also assessed range of doses of donepezil (0.03, 0.1, and 0.3 mg/kg) (Fig. 3b). In both experiments, a group of young mice receiving the vehicle solution was added to assess the effects of aging on performance.

In study A, body weights among the groups ranged from 27.9 ± 3.4 to 33.7 ± 5.1 g, and in study B, they ranged from 28.9 ± 3.5 to 32.4 ± 4.3 g. For the two experiments, no significant between-groups difference was observed (*F*(3,44) < 1.0 and *F*(5,66) < 1.0, respectively). During the food restriction period, both vehicles and drug-treated mice had eaten all their allocated daily amount of dry food. All animals retained for the test session had eaten at least 7/8 pellets out of 10 at both the first and second acquisition sessions.

Acquisition phase. In both studies (A and B), no significant difference was observed among the groups on the total number of head-dips as well as on the % exploration of the baited hole both during the acquisition sessions (*p* > 0.10 in all analyses; data are provided in Suplemental data 1) for both experiments.

#### Test phase

Total number of head-dips. The total number of head-dips was not significantly different among the groups (*F*(5,66) < 1.0). There was no significant effect of either S 47445 or donepezil (*p* > 0.10 in all comparisons versus vehicles) on this parameter for both experiments (data not shown).

Strength of contextual memory (SCM scores). Experiment A (Fig. 3a): A one-way ANOVA showed a highly significant between-groups difference (*F*(4,55) = 10.32; *p* = 0.0001). Analysis of SCM scores showed that middle-aged vehicle-treated mice exhibited a severe memory deficit in remembering the first discrimination (D1) (but not of the second one, data not shown) which was due to an increased amount of interference, compared with young mice (*p* < 0.001).

As regards the effects of S 47445, a one-way ANOVA (young mice excluded from analyses) showed a highly significant between-groups difference (*F*(3,44) = 10.19; *p* = 0.0001). Post hoc Dunnett analysis performed after the one-way ANOVA showed that S 47445 at 0.3 mg/kg (+ 10.57%) produced a higher memory enhancing than S 47445 at 0.1 mg/kg (+ 6.56%) and S 47445 at 0.03 mg/kg (− 0.80%) as compared to middle-aged vehicle-treated group (− 21.66%; *p* < 0.01, *p* < 0.01, and *p* < 0.05, respectively). The gain of performance in S 47445-treated mice on *strength of contextual memory* (SCM) score consisted in a large reduction of interfering responses and a concomitant increase in correct ones.

Experiment B (Fig. 3b): A one-way ANOVA showed a highly significant between-group difference (*F*(6,77) = 13.35; *p* = 0.0001). Analysis of SCM scores showed that middle-aged vehicle-treated mice exhibited a severe memory deficit in remembering the first discrimination (− 33.21%) (but not of the second one, data not shown) compared with young mice (+ 36.58%; *p* < 0.001), thus confirming results of experiment A.

As regards the effects of S 47445 and donepezil, a one-way ANOVA showed a highly significant between-groups difference (*F*(5,66) = 6.28; *p* = 0.0001). Post hoc Dunnett analysis performed after the one-way ANOVA showed that donepezil at 0.3 mg/kg (+ 18.46%) produced a higher memory enhancing than donepezil at 0.1 mg/kg (− 4.04%) as compared to middle-aged vehicle-treated group (− 33.21%; *p* < 0.001 and *p* < 0.05, respectively). In contrast, the lower donepezil dose (0.03 mg/kg; − 17.70%) and S 47445 at 0.01 mg/kg (− 26.41%) and at 0.03 mg/kg (− 19.65%) did not significantly differed from middle-aged vehicle-treated group (*p* > 0.10 in all comparisons).

Regarding all parameters studied, S 47445 at all doses and donepezil at the active doses did not alter memory performance in the second discrimination as compared to middle-aged vehicle-treated mice (data not shown).

### Effect of repeated administration of S 47445 on working memory in a radial maze in aged mice (22–24 months old)

**Fig. 4:**
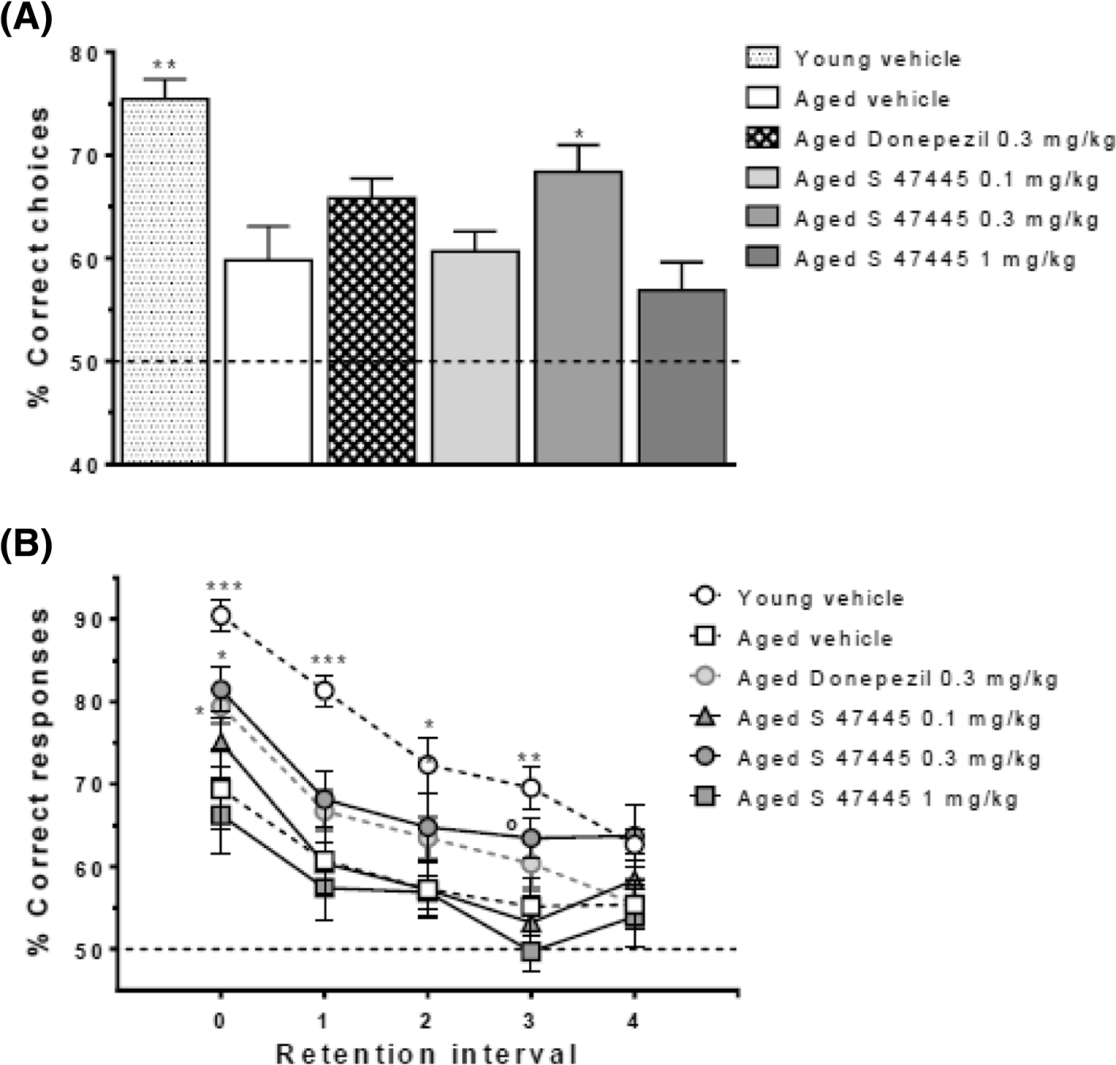
**a**, **b** Effect of S 47445 on radial maze working memory task in aged mice. Effect of repeated administration of S 47445 at the doses of 0.1, 0.3, and 1 mg/kg/day s.c. in aged mice (*n* = 8–12) on the radial arm maze over 12 days. **a** Effect on the percentage of correct choices. Overall mean performance over the 12 days of training (240 trials) are expressed as mean + SEM. *p ≤ 0.05 and **p ≤ 0.01 versus the aged vehicle-injected group (one-way ANOVA followed by Fischer test). **b** Effect on percentage of correct responses as a function of retention interval expressed as mean (over 48 trials for each interval) ± SEM. ^o^ *p* = 0.0524; *p ≤ 0.05; **p ≤ 0.01; ***p ≤ 0.001 versus the aged vehicle group, one way ANOVA followed by Fischer test

In a radial maze working memory task that was designed to assess organizational demand, aged vehicle-treated mice displayed a cognitive deficit as compared to young vehicle-treated mice for overall memory performance and significantly obtained a lower mean percentage of correct responses over the 12 days of training that remained at chance level (50%) when compared to young mice (*p* ≤ 0.01) (*n* = 8–12, Fig. 4a, b). Aged mice treated with S 47445 at 0.3 mg/kg s.c. displayed a better memory performance as compared to aged vehicle-treated mice (*p* ≤ 0.05). A similar effect was observed for donepezil at 0.3 mg/kg s.c. without reaching significance. As expected, when performances were modulated by the retention interval (RI = number of trial interposed; five levels of retention tested from 0 to 4), the mean memory performance over the 12 days decreased in all groups depending on increasing RI (*n* = 8–12, Fig. 4b). In vehicle-treated groups, performance was above chance level for all RI in the young group (at least *p* ≤ 0.001) while performance approached chance level in the aged group above RI = 1 (comparison versus chance level *p* ≤ 0.05 for RI = 0 and RI = 1 but *p* > 0.05 for RI = 2–4) meaning that aged mice failed to remember the previous trial as soon as two intervening trials were interposed. Administration of S 47445 at 0.3 mg/kg reversed aged-induced deficits since their performance remained significantly above chance level regardless the RI level (at least *p* ≤ 0.01). Performance dropped to chance level above R1 = 1 in all other groups except donepezil at 0.3 mg/kg which remained significantly above chance up to RI = 3. A two-way ANOVA revealed a significant effect of treatment (*p* = 0.05) and RI (*p* < 0.0001) with no significant interaction (*p* = 0.549). The lack of significant interaction between treatment and RI was reflecting that the beneficial effect of S 47445 at 0.3 mg/kg was constant across RI (*p* ≤ 0.05 for RI = 0 and *p* = 0.052 for RI = 3). Finally, analyses of treatment effects as a function of proactive inference confirmed that S 47445 at 0.3 mg/kg ameliorated performance whatever the level of proactive interference (data not shown). In contrast, analyses of choice latencies and running times over the 12 training days in the radial maze task (data not shown) failed to show any significant difference between the aged groups. Thus, S 47445 at 0.3 mg/kg statistically improved the aging-related deficit in short-term working memory in the radial maze task. This beneficial effect did not change over training sessions or retention intervals and was significant whatever the organizational demand of the task.

To summarize the findings of experiment 1, data drawn in the CSD task showed that the two higher doses of donepezil (0.1 mg/kg and 0.3 mg/kg) but not the lower dose (0.03 mg/kg) induced a memory-enhancing effect in middle-aged mice. The dose of donepezil at 0.3 mg/kg also significantly reduced age-related memory decline in the radial arm maze task. In addition, whereas the low doses of S 47445 (0.01 mg/kg and depending on the experiments the dose of 0.03 mg/kg) presented no memory-enhancing effect in the CSD task, a significant memory-enhancing effect of S 47445 has been observed at 0.1 and 0.3 mg/kg and in the radial arm maze at 0.3 mg/kg. Thus, given these results, the inactive lower (0.03 mg/kg) and intermediate (0.1 mg/kg) doses of donepezil were chosen for subsequent pharmacological experiments with S 47445. Given the lack of memory-enhancing effect of the dose of 0.01 mg/kg, the variable effect of 0.03 mg/kg of S 47445 in the CSD task, and the memory-enhancing effect observed at a higher dose (0.3 mg/kg) in both the CSD and the radial arm maze task, we have chosen the doses 0.03, 0.1, and 0.3 mg/kg of S 47445 for the combination study in experiment 2.

### Experiment 2: effects of S 47445 administered alone or combined with donepezil on CSD and SA rates in middle-aged (CSD task) and aged mice (SA rates)

In experiment 2, pharmacological compounds in combination or alone for comparison were administered per os according to the procedure described for the CSD task of experiment 1. In addition to the CSD task, independent groups of 18–19-month-old mice have been submitted to a SA in order to keep on studying working memory (Faucher et al. 2016). Indeed, we have already shown that working memory impairments are observed in the SA task in 17/18-month-old animals as compared to young adult (4–5 months old) or middle-aged (14–15 months old) mice and that the SA task can be useful to evaluate procognitive compounds in aged animals (Vandesquille et al. 2011, 2013).

#### Contextual memory in the CSD task in middle-aged mice (14–15 months old)

According to data drawn in experiment 1, the two doses of donepezil 0.03 and 0.1 mg/kg (respectively, Don1 and Don2) were combined with doses of S 47445 (0.03, 0.1, and 0.3 mg/kg; respectively S1, S2, and S3). The study was carried out on 12 groups receiving either compound alone (vehicles, Don1, Don2, S1, S2, and S3) or their combinations (Don1 + S1, Don1 + S2, Don 1 + S3, Don2 + S1, Don2 + S2, and Don2 + S3), with *N* = 12 mice per group.

#### Acquisition phase (data not shown)

No significant difference was observed among the groups as regard the total number of explorations of the four holes (*p* = 0.67). The evolution (increase or decrease) of the total number of exploration of the four holes from the first to the second acquisition session was not significantly different (*p* = 0.34). Moreover, the interaction “treatments × discriminations” was not significant (*p* = 0.35).The percent number of exploration of the baited hole was not significantly different among the groups (*p* = 0.81). The evolution of scores from acquisition session 1 to acquisition session 2 as shown by repeated measures ANOVA was not significant (*p* = 0.26) as well as the interaction between treatments and discriminations (*p* = 0.08).

#### Test session

**Fig. 5:**
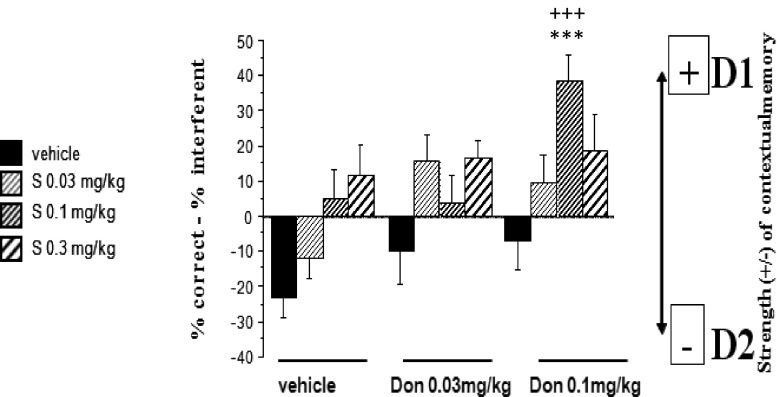
Synergistic effect of S 47445 and donepezil on SCM scores in middle-aged mice. SCM scores are expressed as means ± SEM. SCM scores are obtained by calculation (% of correct responses − % of interfering responses). Only the synergistic effect is represented in this figure. Indeed, even though all S 47445 dose administered with donepezil at 0.1 mg/kg produced significant improvement of SCM scores as compared to each compound alone (see Table 1 for detailed comparisons), the greater and highly significant synergistic memory-enhancing effect was observed with the combination of the intermediate S 47445 dose (0.1 mg/kg) combined with donepezil (0.1 mg/kg) (versus donepezil alone: ***p < 0.001), versus S 47445 alone (+++*p* < 0.001)

No significant interaction between donepezil and S 47445 on SCM scores was revealed (*p* = 0.131); thus, the dose effect of S 47445 or donepezil has been analyzed at all pooled levels of the other drug. Data are represented in Table 1 and Fig. 5.

**Table 1:** Effects of S 47445 and donepezil, alone or in combination on SCM scores in middle-aged (14–15 months old) in the CSD task

Donepezil	All levels of S 47445	S47445			
		Vehicle	0.03 mg/kg	0.1 mg/kg	0.3 mg/kg
All levels of donepezil			+	+++	+++
Vehicle		− 23.1020 ± 7.7919	− 12.5242 ± 7.7919	5.1515 ± 7.7919	11.7033 ± 7.7919
0.03 mg/kg	NS (*p* = 0.079)	− 10.0898 ± 7.7919	15.8591 ± 7.7919	3.8137 ± 7.7919	16.6148 ± 7.7919
0.1 mg/kg	***	− 7.4153 ± 7.7919	9.6217 ± 7.7919	38.3934 ± 7.7919	18.7178 ± 7.7919

SCM scores are expressed as means ± SEM. SCM scores are obtained by calculation (% of correct responses − % of interfering responses). Results are expressed as LSMeans ± SE LSMeans

Post hoc analysis of donepezil effect for all S 47445 levels with Dunnett test: comparisons versus vehicle group; NS *p* > 0.050; *p ≤ 0.050; **p ≤ 0.010; ***p ≤ 0.001. Post hoc analysis of S 47445 effect for all donepezil levels with Dunnett test: comparisons versus vehicle group; NS *p* > 0.050; +*p* ≤ 0.050; ++*p* ≤ 0.010; +++*p* ≤ 0.001

Two-way analyses of variance showed a significant effect of S 47445 (*p* < 0.001). As compared to pooled levels of donepezil, all doses of S 47445 increased significantly SCM scores (*p* < 0.05, *p* < 0.01, and *p* < 0.001 respectively) as shown in Table 1.

As shown in Fig. 5, all the doses of S 47445 (0.03, 0.1, and 0.3 mg/kg) combined with donepezil at 0.1 mg/kg produced a significant improvement of SCM scores as compared to both compounds administered alone at the same dose as observed in the post hoc analyses (for donepezil effect for all S 47445 levels: *p* < 0.001 for donepezil at 0.1 mg/kg with all the tested doses of S 47445 and conversely, S 47445 effect for all donepezil levels p < 0.05; p < 0.001 and p < 0.01, respectively for donepezil at 0.1 mg/kg with all the tested doses of S 47445, versus respective vehicle group).

#### Analysis of dose effect of donepezil at pooled levels of S 47445

The dose effect of donepezil has been analyzed at pooled levels of S 47445. Two-way analyses of variance showed a significant effect of donepezil (*p* = 0.02). As compared to vehicle, donepezil at 0.1 mg/kg increased significantly SCM scores (*p* = 0.001) whereas a tendency was observed for the dose of donepezil at 0.03 mg/kg (*p* = 0.079; see also Table 1).

Regarding all parameters studied, S 47445 at all doses and donepezil at the active dose did not alter memory performance in the second discrimination as compared to vehicle-treated mice. Indeed, the pharmacological treatments, either compounds alone or combined, have no effect on the memory retrieval of the second discrimination. This finding was of interest in so far as the memory-enhancing effect of the compounds alone or combined on D1 could have resulted in an increase of proactive interference and to a concomitant decrease of the memory of the second discrimination D2, leading therefore to an inverse memory retrieval pattern as compared to vehicle-treated mice. Thus, it can be assumed that the pharmacological treatments alone or combined did not show any adverse memory effect on memory retrieval curve in middle-aged mice (supplemental data 2).

### Working memory in the SA task in aged mice (18–19 months old)

#### Alternation over the seven trials of the series

This study was conducted using donepezil at 0.1 mg/kg only. Data are summarized in Table 4. When considering the percentage of alternation calculated over the seven first trials (all spaced by a 90-s delay), global statistical analysis showed (i) that S 47445 increased the level of alternation (*F*(5132) = 6.357; *p* < 0.0001) as well as donepezil (0.1 mg/kg) (*F*(1132) = 15.6; *p* = 0.0001) and (ii) the interaction between donepezil and S 47445 is also significant (*F*(5132) = 3.81; *p* = 0.0029). Further analysis with the Dunnett’s test indicated that the group treated with S 47445 at 0.1 mg/kg or donepezil 0.1 mg/kg alone alternated significantly above vehicles (*p* < 0.05 in both cases). In addition, the combination of S 47445 at 0.3 and 0.1 mg/kg and donepezil induced a significant improvement of alternation rates as compared to vehicles (*p* < 0.001 and 0.01, respectively) whereas only the combination of S 47445 at 0.3 mg/kg and donepezil increased significantly performance as compared to either S 47445 at 0.3 or donepezil alone (*p* < 0.01 in both comparisons). Differences of other doses, alone or in combination, versus the control group were not significant (Table 2).

**Table 2:** Effects of S 47445 and donepezil, alone or combined on SA rates in aged (18–19 months old) animals

Donepezil	S47445					
	Vehicle	0.01 mg/kg	0.03 mg/kg	0.1 mg/kg	0.3 mg/kg	1.0 mg/kg
Vehicle (0.0 mg/kg)	48.611 ± 5.603 *n* = 12	59.722 ± 2.477 *n* = 12	61.11 ± 4.738 *n* = 12	65.278 ± 3.815 *n* = 12 *	61.111 ± 2.369 *n* = 12	59.722 ± 3.217 *n* = 12
0.1 mg/kg	65.218 ± 3.815 *n* = 12 *	58.333 ± 2.513 *n* = 12	62.500 ± 4.167 *n* = 12	76.389 ± 4.322 *n* = 12 *	86.11 ± 3.453 *n* = 12 */++	59.722 ± 4.332 *n* = 12

Alternation scores are calculated from data issued from the seven first trials of the series (intertrial interval 90 s) and are expressed in percentage. *p < 0.05 versus vehicles; ++*p* < 0.01 versus compound (S 0.3 mg/kg and Don 0.1 mg/kg) alone

Statistical analysis evidenced a significant increase of alternation rates at the 8th trial of the series as compared to the 7th one in all groups (all paired “*t*” test < 0.05) so that no between-group difference on the percentage of alternation at the 8th trial of the series was observed (*p* > 0.05). In addition, the interaction between donepezil and S 47445 was also not significant (*F*(5, 132) = 0.879; *p* = 0.49). Thus, an alteration of the motivation to alternate cannot be ascribed as responsible of the deficit in middle-aged mice and the treatments did not alter the alternation behavior.

### Alternation per block of trials in the series

As interference increases over the series of trials, an analysis per blocks of trials (i.e., block 1: % trials 2 + 3; block 2: % trials 4 + 5; block 3: % trials 6 + 7) was conducted. An analysis per blocks of two consecutive trials evidenced no significant interaction between S 47445 and donepezil whatever the block of trials considered (*p* > 0.13 in all analyses). A significant difference was observed on the third block (trials 6–7; *p* = 0.0008; Table 3) but not on the second block (trials 4–5; *p* = 0.063; data not shown). In addition, a significant S 47445 effect was observed on block 2 (*p* = 0.027) and block 3 (*p* = 0.0003).

**Table 3:** Effects of S 47445 and donepezil, alone or in combination on SA rates at the 3rd block of trials in aged (18–19 months old) animals

Donepezil	S47445					
	Vehicle	0.01 mg/kg	0.03 mg/kg	0.1 mg/kg	0.3 mg/kg	1.0 mg/kg
Vehicle	50.000 ± 6.155 *n* = 12	45.833 ± 9.650 *n* = 12	50.000 ± 8.704 *n* = 12	70.833 ± 7.432 *n* = 12	50.000 ± 8.704 *n* = 12	58.333 ± 8.333 *n* = 12
0.1 mg/kg	45.833 ± 4.167 *n* = 12	41.667 ± 12.050 *n* = 12	45.833 ± 11.445 *n* = 12	87.500 ± 6.528 *n* = 12 *	83.33 ± 7.107 *n* = 12 *	54.167 ± 9.650 *n* = 12

Alternation scores are calculated using data from the two last (6 + 7) trials of the series (intertrial interval 90 s) and are expressed in percentage. The combination of donepezil and S 47445 at 0.1 and 0.3 mg/kg induced a significant reduction of interference and increased SA rates as compared to vehicles (*p < 0.05 versus vehicles)

Interestingly, the “Don 0.1 + S 47445 at 0.1 mg/kg” and “Don 0.1 + S 47445 at 0.3 mg/kg” groups emerged as the more powerful combinations to reduce proactive interference over the series as compared to vehicles.

### Irwin and pharmacokinetic study

S 47445 administered alone is devoid of any observable effect in the Irwin test over the dose range 3 to 100 mg/kg p.o. Donepezil administered alone had clear and dose-dependent sedative/myorelaxant effects over the dose range 3–10 mg/kg p.o. in the same test. Tremor, hypothermia, and decreases in activity, abdominal muscle tone, and reactivity to touch were observed at 3 mg/kg p.o. and which were accompanied by Straub tail and abnormal gait (rolling) at 10 mg/kg p.o. When administered in combination, S 47445 (3–100 mg/kg p.o) did not modify the effects of donepezil (0.3–10 mg/kg p.o.) suggesting no detrimental additive effects shown with S 47445across the selected dose range.

Pharmacokinetic results are summarized in Table 4. After repeated administration of S 47445, the interindividual variability on S 47445 blood concentrations was moderate to high. C_max_ was reached between 1 and 4 h after dosing. The apparent elimination half-life was ~ 2.1 to 3.6 h. There was no evidence of a change in S 47445 blood exposure when administered with donepezil. Moreover, after repeated administration of donepezil, the interindividual variability on donepezil plasma concentrations was high. C_max_ was reached 0.5 h after dosing. The apparent elimination half-life was around 2 h. There was no evidence of a change in donepezil plasma exposure when administered with S 47445. Tmax was not changed following combinations of the drug (t_max_ for S 47445 = 1.5 h and t_max_ for donepezil = 0.5 h).

**Table 4:** Mean pharmacokinetic parameters of S 47445 (0.3 mg/kg) and donepezil (1.0 mg/kg), given alone or combined in middle-aged mice

	S 47445 (0.3 mg/kg)		Donepezil	
Parameter	Alone	With donepezil (1 mg/kg)	Alone	With S 47445 (0.3 mg/kg)
Mean AUClast (ng h/mL) [% CV]	32.1 [7.83]	32.6 [23.0]	24.0	21.4
Mean Cmax (ng/mL) [% CV]	10.2 [13.7]	8.46 [20.8]	18.5	12.0
Median Tmax (h) [range]	1.5 [1–4]	1.5 [1–2]	0.5	0.5
T1/2z (h) [% CV]	2.09 [32.0]	3.58 [66.9]	1.74	2.15

Three groups (*n* = 4 subjects for S 47445 and *n* = 4 per sampling time for donepezil) were used for the pharmacokinetic (PK) study

## Discussion

Effects of donepezil and S 47445 administered alone or in combination on age-induced memory deficits

The present results provide evidence that S 47445 given alone possesses cognitive-enhancing properties after repeated administration in two different tasks based on aging-induced memory deficits in mice, the CSD, and a working memory model using radial maze.

The cognitive impairments observed in the present study in middle-aged and aged animals are in accordance with our previous studies, showing that young adult 4–5-month-old mice exhibited significant and substantial memory performance in those tasks, as compared to middle-aged and aged animals (Béracochéa et al., 2007; Marighetto et al. 2008a, b; Mingaud et al. 2008; Vandesquille et al. 2011; Sors et al. 2017). In fact, middle-aged mice showed a negative SCM score on the retention of D1 in the CSD task that indicated an increase of interfering responses, i.e., an increase of head-dips into the baited hole of the second acquisition during memory testing of the first one. Such an increase in interfering responses is also observed in aged mice which exhibited an exaggerated vulnerability to interference over the series or as a function of the memory load of the task in both the SA and radial arm maze tasks, respectively.

Whereas procognitive effects of AMPA-PAMs have been described in a variety of models (Bernard et al. 2010; Morrow et al. 2006; Lynch and Gall 2006; O’Neill and Dix 2007; Partin 2015), few studies have investigated their effects after repeated dosing. In the present study, repeated administration of both compounds given alone from 0.03 to 0.3 mg/kg (S 47445 or donepezil) dose-dependently reversed aging-related memory impairments, as those mice exhibited in the CSD task a substantial memory of D1 as compared to vehicle-treated animals. In addition, these compounds also reduced significantly the age-induced vulnerability to interference in the radial arm maze and in the alternation tasks. Interestingly, the way to administrate the drugs (per os in the CSD task or s.c. in the radial arm maze task) does not interfere with the efficacy of the compounds administered alone, since both types of administrations revealed similar procognitive active doses for donepezil and S 47445. These data lead us to choose the chronic oral administration for the combination study, since this route is the one use for donepezil and the anticipated route of use of S 47445 in humans. Repeated s.c. administration of S 47445 at 0.3 mg/kg demonstrated beneficial effects against the aging-related degradation of short-term/working memory using radial maze. Unrelated to the training level, retention interval, and organization demand, S 47445 can improve globally working memory both in short-term retention of varying information and in organization of information. This task was proposed as a model of everyday life memory requiring both temporary retention of numerous ever-changing information and organization/update of this information to avoid interference from irrelevant memories (Al Abed et al. 2016). In that sense, global efficiency of S 47445 at 0.3 mg/kg against the age-related deficit observed here is of noticeable interest. In comparison, activity of donepezil at 0.3 mg/kg i.p. was limited to the organizational component of the task (Marighetto et al., 2008b). The apparent limitation of S 47445 dose range effect restricted to 0.3 mg/kg echoes the effect of donepezil using the same working memory task where the dose of 0.3 mg/kg but not of 1 mg/kg was found to be active (Marighetto et al., 2008b). The efficacy of S 47445 was also confirmed after oral repeated treatment in the contextual and serial discrimination task. In this model, middle-aged mice exhibit specific internal contextual memory impairment but no spatial memory deficit (based on a correct use of external contextual cues), which may represent an early form of age-related deficit. Herein, S 47445 (0.1 and 0.3 mg/kg p.o.) reversed the age-induced deficits by acting both on correct responses and interfering responses in the same range of doses. Thus, it indicated the recovery of use of flexible episodic-like form of memory in treated middle-aged mice (Celerier et al., 2004; Tronche et al., 2010). S 47445 showed comparable efficacy to the cholinesterase inhibitor donepezil in contextual and serial discrimination task (Béracochéa et al., 2007).

Further, it was of particular interest to determine potential synergistic memory-enhancing effects of S 47445 and donepezil administered in combination on memory in middle-aged and aged animals. Indeed, as regards the memory retrieval of the first discrimination in the CSD task, combinations of donepezil at the lowest dose (0.03 mg/kg) and S 47445 at all doses have a significant effect, even though mild (*p* < 0.05) on SCM scores, as compared to donepezil or S 47445 alone; a similar conclusion can be drawn from the combination of the higher S 47445 dose (0.3 mg/kg) and the two doses of donepezil (see Table 1). In contrast, the combination of the highest donepezil dose (0.1 mg/kg) and the intermediate S 47445 dose (0.1 mg/kg) produced the main procognitive effect (*p* < 0.01 in both analyses) in comparison to the other drugs combinations and also induced the higher level of interference on the retention of D2 (supplemental data 2), thus confirming its efficacy in improving memory of D1. Collectively, combination of donepezil and S47445 dose-dependently can induce a synergistic procognitive effect.

The pharmacological treatments, either compounds alone or combinations had no effect on the memory retrieval of the second discrimination (supplemental data 2), with few exceptions that could not be considered as reflections of negative side effect. This finding was of interest in so far as the memory-enhancing effect of the compounds alone or combined on D1 could have resulted in an increase of proactive interference and to a concomitant decrease of the memory of D2, leading therefore to an inverse memory retrieval pattern as compared to vehicle-treated mice. Thus, it can be assumed that the pharmacological treatments alone or combined did not show any adverse memory effect on memory retrieval curve in middle-aged mice.

A similar conclusion can be drawn from the SA task in aged mice. Regarding the percentage of alternation, the interaction between S 47445 and donepezil was significant (*p* = 0.00029), indicating that the dose effect of S 47445 on the percentage of alternation depends on the dose of donepezil ( and 0.1 mg/kg) and inversely. Interestingly, the combination of donepezil and S 47445 at 0.3 mg/kg have a significant effect on the % of alternation as compared to either S 47445 at 0.3 mg/kg or donepezil alone (*p* < 0.01 in both cases). Moreover, the combination of S 47445 at 0.3 mg/kg and donepezil protects from the deleterious effects of proactive interference over the series in the SA task mainly during the third blocks of trials.

As the two compounds were administered simultaneously, it was important to assess a potential pharmacokinetic interaction, which could have faked a synergistic effect by increasing the blood exposure. Our study confirmed that the synergistic effect of the combination of the two compounds is not due to a pharmacokinetic interaction between them. Moreover, the safety of S 47445 and donepezil, alone or in combination, was evaluated using the primary observation (Irwin) test in mice and results showed that the combination of pharmacological doses of both compounds did not modify the sedative/myorelaxant effects of donepezil, and reveal no other behavioral aspects similarly as compounds administered alone.

To sum up, our study emphasized that both S 47445 alone and donepezil alone have a memory-enhancing effect in aged animals both in the CSD and SA tasks. However, combining donepezil (0.1 mg/kg) with S 47445 mainly at the dose of 0.1 mg/kg (CSD) or 0.3 mg/kg (SA) creates a synergistic effect on memory performance which revealed a stronger memory-enhancing effect as compared to compounds alone.

Our data are in agreement with other pharmacological studies in rodent’s models or in AD patients, showing the benefits of combinations of donepezil with drugs acting on other neurotransmission systems such as histaminergic neurotransmission (Cho et al. 2011) or NMDA receptors (Ota et al. 2015; Atri et al. 2015). However, these studies (at the exception of Sors et al. 2017) demonstrated additive effects of the combinations of the drugs (which means that the effect of two compounds is equal to the sum of the effect of the two compounds taken separately) rather than a synergistic interaction, as this is the case of our present study. Thus, our study provides original and clear-cut evidences of such a synergistic interaction between these two compounds, moreover in mice models of natural age-induced memory dysfunction.

### Hypothesis on the mechanisms of action

We already performed immunohistochemical analyses on the effects of aging on CREB phosphorylation (pCREB) used as a marker of neural activity either in middle-aged mice after CSD memory testing or in aged mice after alternation testing. We showed more precisely that middle-aged animals exhibited after CSD testing significant reductions of pCREB in the CA1 of the dorsal and ventral hippocampus and in the medial septum as compared to young vehicles (Sors et al. 2017). In aged mice, the alternation deficit observed was associated with a significant reduction of pCREB in the prelimbic cortex and more moderately in the hippocampus (Vandesquille et al. 2013). Thus, aging induced an alteration of the functioning of the prefrontal-cortex-hippocampus network known to be critically involved in contextual and working memory processes (Teles-Grilo Ruivo et al. 2017). This assertion is also confirmed through experiments conducted in previous studies. Indeed, we showed that lesioning or inactivating the dorsal hippocampus totally suppressed memory of D1 (Chauveau et al. 2009; Dominguez et al., 2016) whereas lesioning or inactivating the prelimbic cortex suppressed memory of D2 (Chauveau et al. 2008; Dominguez et al., 2016).

S 47445 is a potent facilitator of AMPA glutamatergic receptors and that glutamatergic neurotransmission is altered in aged or in AD patients (Hascup et al. 2016; Jin et al. 2017), whereas donepezil is a well-known anticholinesterase compound which increases the efficacy of the cholinergic neurotransmission (Scali et al. 2002). Little is known about AMPA receptor expression during aging. In pathologic aging, such as in AD, reports showed a decrease of AMPA binding sites in AD brain (Armstrong et al. 1994) as well as a dramatic reduction of NMDA and AMPA receptors at the postsynaptic density (Gong et al. 2009). More recently, it was suggested that the subunits content of AMPA receptors GluA1–GluA4 at the membrane may be an important factor for cognitive prospect. Indeed, Yang et al. (2015) have shown that rats with cognitive impairment in the Morris Water Maze presented a much lower surface expression of GluR1 in the hippocampus than young adults and aged rats with preserved cognitive abilities. Given the previous findings on pCREB activity in the hippocampo-frontal circuitry, it can be proposed that the enhancement of memory performance resulting from the combination of S 47445 and donepezil could be due to a higher facilitation of glutamatergic and/or cholinergic activities in this network, as compared to donepezil or S 47445 alone. Within this framework, the combination of the two compounds could exert a synergistic beneficial action on the activity of the prefrontal cortex-hippocampus network which alterations are involved in the memory decline observed in aged or AD patients.

### Conclusion

The present study shows that the potent and selective AMPA-PAM S 47445 demonstrated to be active alone in improving cognitive performance after repeated administration in two different memory models, the radial maze task and assessing contextual memory performance, both sensitive to aging and assessing either episodic-like or working memory. Further, the combination of the two memory-enhancing compounds, donepezil and S 47445, can lead to synergistic memory-enhancing effects in middle-aged and aged mice either on episodic-like or working memory, with a statistically higher cognitive impact never obtained with any memory-enhancing compound alone, and without any pharmacokinetic interaction between both compounds. Altogether, combining different drug mechanisms can have a therapeutic interest for treating cognitive deficits as those associated with Alzheimer’s disease.

## Electronic supplementary material

ESM 1 (DOCX 139 kb)
