# Supplementary material for: Synergistic enhancing-memory effect of donepezil and S 47445, an AMPA positive allosteric modulator, in middle-aged and aged mice
Source: Psychopharmacology (Berl). 2017 Nov 22;235(3):771–87. doi: 10.1007/s00213-017-4792-5 (PMC5847048; doi:10.1007/s00213-017-4792-5)
Supplement: Supplementary file 1 — (DOCX 139 kb) [file 213_2017_4792_MOESM1_ESM.docx]

**Supplemental data 1.**

**Table 1. Effects of S 47445 and Donepezil, administered alone, on the total number of explorations and the % exploration of the baited hole at the first and second discrimination of the acquisition phase of Experiment 1 (Study A and Study B).** No significant between-groups difference was observed on both parameters (p> 0.10 in all analyses).

**
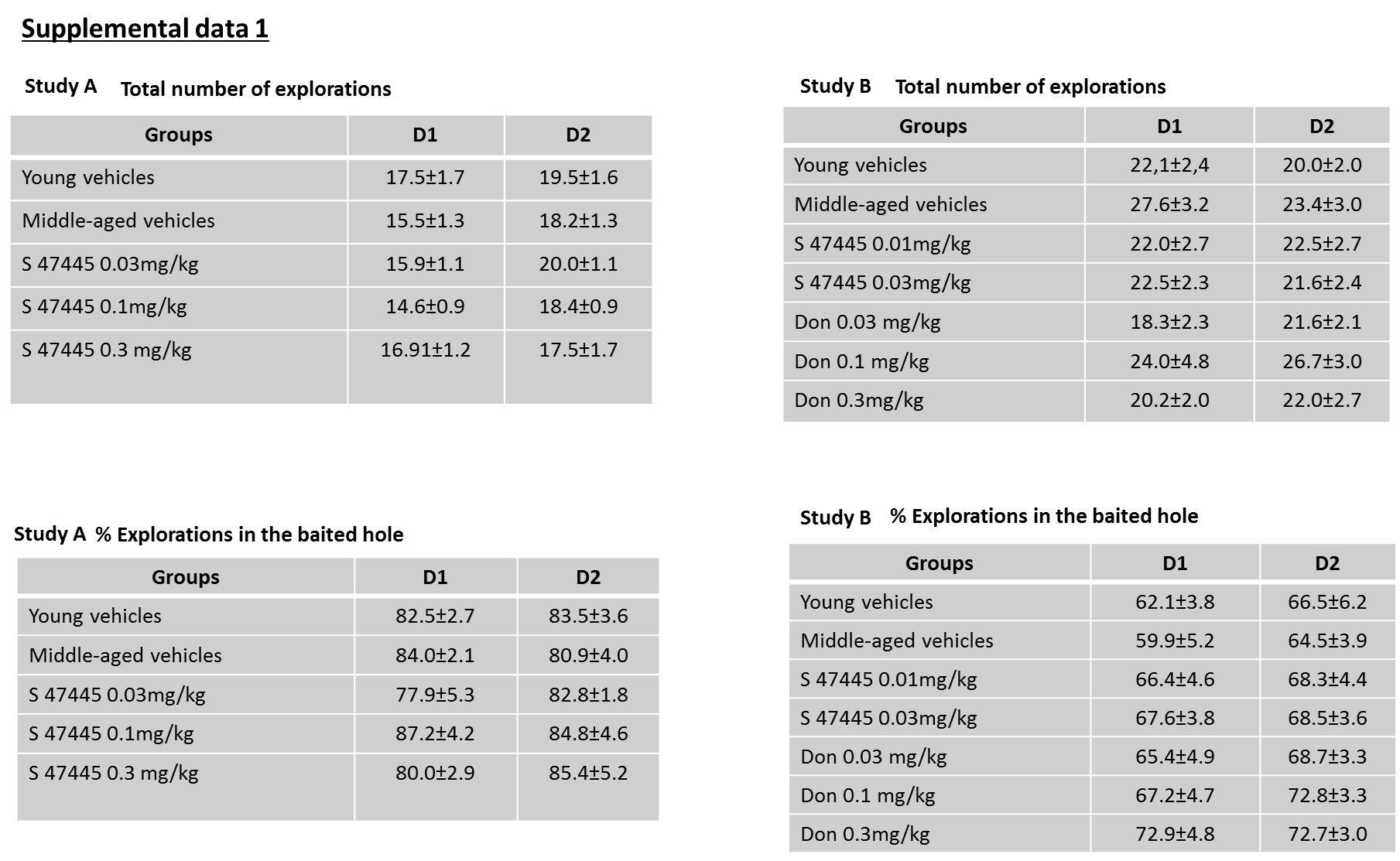
**

**Supplemental data 2.**

**Effects of S 47445 and Donepezil, alone or combined, on SCM score of the second discrimination in middle-aged mice in the CSD task.**

**Rationale:** to study the effects of Donepezil at 0.03 and 0.1 mg/kg and S 47445 at 0.03; 0.1; 0.3 mg/kg p.o., alone or combined on memory of the second discrimination in the CSD task. As for memory testing of discrimination 1 in Experiment 2, the difference between the % of correct responses and the % of interferent responses (“% correct responses - % interferent responses” = SCM scores) was calculated. Indeed, during the test session, interferent responses reflect the memory of the baited hole at the first discrimination (D1) of the acquisition phase whereas correct responses reflect the memory of the hole baited at the second discrimination (D2). The higher is the difference between the % of correct responses and the % of interferent responses, the higher is the strength of contextual memory.

**Material and Methods**: Animals, behavioral experiments, and drugs administration (chronic *per os* treatment) were as those described in Experiment 2 in the manuscript. For this study, there were 12 groups of 12 mice each. The different groups were as follows: 1) Vehicle (1% (w/v) HEC and 1% (v/v) polysorbate 80 in distilled water); 2) Donepezil: 0.03 mg/kg (Don1); 3) Donepezil: 0.1 mg/kg (Don2); 4) S 47445 0.03 mg/kg p.o. (S1); 5) S 47445 0.1 mg/kg p.o. (S2); 6) S 47445 0.3 mg/kg p.o. (S3); 7) Don 1 + S1; 8) Don1 + S2; 9) Don1 + S3; 10) Don 2 + S1. (S4); 11) Don 2 + S2; 12) Don2 + S3. Drug administration was as follow: day 1 to day 7: one *per.os* administration/day of either S 47445 or Donepezil or combined (both compounds in one solution only, extemporaneously). Day 8: administration 1h before acquisition, mice learned two discriminations (D1 and D2); Day 9: administration 1h before retention testing of 1^st^ discrimination D1.

**Results:**

**Acquisition session**.

The treatments did not induce significant differences among groups for the total number of explorations of the 4 holes and the number of explorations of the baited hole at acquisition 1 and 2 (p>0.10 in all analyses; data not shown). Thus, it can be assumed that any difference observed between the groups at test session cannot be ascribed to side negative effects of either ageing or of the pharmacological treatments on exploratory patterns or motivation for food at the acquisition phase.

**Test session.**

The interaction (S 47445 x Donepezil) was not significant (p= 0.163). Thus, the dose effect of S 47445 or Donepezil has been analyzed all levels pooled of the other drug. There was no significant effect of S 47445 (p=0.951) as well as Donepezil (p=0.074) on the strength of contextual memory responses. Data are represented in Figure 2 supplemental data A and B.

**
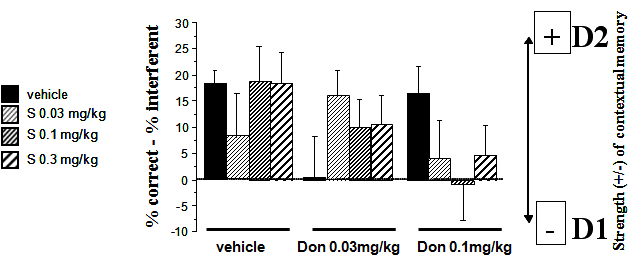
**

A

**
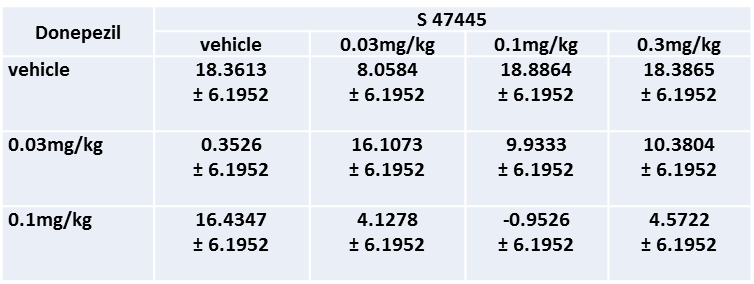
**

B

**Figure . supplemental data2. Effects of S 47445 and Donepezil, alone or in combination, on SCM score of the second discrimination in middle-aged mice in the CSD task.** Results are expressed as LSMeans ± S.E. LSMeans.

**Conclusion**. On D2, the interaction between S 47445 and Donepezil was not significant. Thus the dose effect of S 47445 was analyzed at pooled levels of Donepezil, and inversely. Vehicle-treated mice showed a significant memory of D2 and both S 47445 and Donepezil alone or in combination, did not significantly modified SCM scores as compared to vehicles (NS in all comparisons). Interestingly, the combination of Donepezil at 0.1 mg/kg and S 47445 at 0.1mg/kg which induced the higher retention of D1 in Experiment 2 (see manuscript), also produced the higher amount of interference in the present experiment on D2, thus confirming indirectly that this combination is efficacious in improving memory of D1.
